# Recent Legislation on Criminal Lunatics

**Published:** 1860-10-01

**Authors:** 


### Art. IX.—RECENT LEGISLATION ON CRIMINAL LUNATICS.

An Act of the last session (23 and 24 Vic., cap. 75) makes further provision for the custody and care of "Criminal Lunatics." The persons to whom it refers by this title are comprised in the following classes:—viz.: 1. Prisoners who on trial have been acquitted on the ground of insanity; 2, prisoners who upon arraignment have been found to be insane; 3, convicts in Pentonville or Millbank prison becoming or found insane during confinement, or any convict under sentence of penal servitude.

Section 1 provides that it shall be lawful for Her Majesty to appoint that any asylum or place in England which Her Majesty may deem suitable for this purpose shall be an asylum for criminal lunatics, and that the provisions of the Act shall be applicable to every such asylum.

Section 2 provides that it shall be lawful for one of Her Majesty's principal Secretaries of State to direct to be removed to and kept in any such asylum any person coming under either of the above-mentioned classes, including any person who under any previous order may have been placed in any county lunatic asylum, or other place of reception for lunatics: and that with every person removed under any such order there shall be transmitted a certificate, as set forth in schedule A, duly filled up and authenticated, the contents of which certificate shall be transcribed into the general register to be kept in every such asylum.

Section 3 provides that nothing in the Act shall affect the authority of the Crown to make other provision for the custody of a criminal lunatic.

Section 4 empowers the Secretary of State to appoint any persons he may think fit, not being less than three in number, to be a council of supervision for any asylum under the Act; and also to appoint for the asylum a resident medical superintendent, a chaplain, and such other officers, assistants, and servants as he may deem necessary; and the Secretary of State, with the approval of the Commissioners of Her Majesty's Treasury, is to fix the salaries to be paid to the superintendent, chaplain, officers, assistants, and servants of such asylum. From this latter provision, it would appear that the council of supervision is intended to be an unpaid board.

Section 5 empowers the Secretary of State to make rules for the government of the asylum; and section 6 directs that, subject to such rules, the council of supervision shall superintend the asylum.

Sections 7 and 8 relate to the removal and discharge of prisoners. Under the latter, convicts whose term of imprisonment or penal servitude has expired may be discharged, although not certified to have become of sound mind, to the intent that they may be placed in a county lunatic asylum, or otherwise subjected to the same care and treatment as lunatics not being criminals.

Section 9 enables the Secretary of State to permit any person confined in the asylum to be absent from such asylum upon such conditions, in all respects, as to the Secretary of State shall seem fit; and provides that any person absent after any such condition broken, may be retaken as in case of an escape.

Section 11 provides, in case of escape, for the recapture of lunatics by the superintendent, or any officer or servant of the asylum, or any person assisting them, or any person authorized in writing by the Secretary of State or the superintendent of the asylum.

Section 12 relates to the punishment of persons for rescue or for permitting escape.

Section 13 contains penalties for illtreating lunatics.

Section 14 directs the visitation of the asylums by the Commissioners in Lunacy; and section 15 directs them to report thereon to one of Her Majesty's Secretaries of State, in the month of March, every year.

